# The number of previous implantation failures is a critical determinant of intrauterine autologous platelet‐rich plasma infusion success in women with recurrent implantation failure

**DOI:** 10.1002/rmb2.12565

**Published:** 2024-02-29

**Authors:** Shunsaku Fujii, Takaaki Oguchi

**Affiliations:** ^1^ ef.clinic Aomori Japan

**Keywords:** embryo implantation, embryo transfer, endometrium, infertility, platelet‐rich plasma

## Abstract

**Purpose:**

We aimed to identify factors influencing the reproductive outcomes of frozen–thawed embryo transfer (FET) with intrauterine autologous platelet‐rich plasma (PRP) infusion in patients with either a thin endometrium or recurrent implantation failure (RIF) despite a normal endometrial appearance.

**Methods:**

In this retrospective study of women who underwent PRP‐FET, factors influencing PRP‐FET outcomes were identified using multivariate logistic regression analysis.

**Results:**

We enrolled 111 patients (70 with refractory thin endometrium and 41 with RIF but no thin endometrium). For 99 completed FET cycles, the β‐hCG positivity rate was 46.7%, clinical pregnancy rate (CPR) was 41.0%, and live birth rate (LBR) was 36.2%. PRP treatment was associated with significant improvements over previous cycles, and participants with thin endometria demonstrated thickening. Multivariate logistic regression analysis showed that the number of previous implantation failures in women with RIF was a significant factor affecting the PRP‐FET outcomes. The CPR and LBR of women with RIF were lower when there had been ≥3 previous implantation failures occurred.

**Conclusions:**

Intrauterine PRP infusion improves the pregnancy outcomes of patients with RIF or a thin endometrium. The number of previous implantation failures is a critical determinant of successful intrauterine PRP infusions in women with RIF.

## Graphical abstract

Intrauterine PRP infusion improves the pregnancy outcomes of patients with RIF or a thin endometrium. The number of previous implantation failures is a critical determinant of the success of intrauterine PRP infusion in women with RIF.

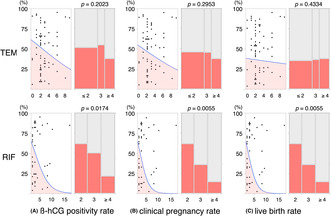

## 1 INTRODUCTION

Implantation failure refers to a situation in which a woman undergoing assisted reproductive technology (ART) fails to become pregnant, despite the transfer of high‐quality embryos. The pathophysiology of implantation failure is multifactorial, and recurrent failure (RIF) poses a significant challenge in ART, because it is associated with prolonged physical, emotional, and financial burdens for the patients. However, there is a lack of consensus regarding the precise definition of RIF. While both uterine and embryonic factors are generally recognized to be critical contributors to RIF, endometrial receptivity plays a pivotal role in successful embryo implantation, which is underscored by the implantation rate of euploid blastocysts being <70%.^1^

A thin endometrium is considered a uterine factor that contributes to implantation failure. A thin endometrium is not only associated with repeated cycle cancellation, but is also linked to lower pregnancy rates, spontaneous abortion, ectopic pregnancies, abnormal placentation,^2^, ^3^ and obstetric complications.^4^, ^5^ However, even for patients with a thin endometrium but no anatomical defects, there are few available adjuvants for treatment. Current recommendations, such as long‐term or high‐dose estrogen administration, low‐dose aspirin, vitamin E supplementation, vaginal sildenafil citrate application, and the intrauterine infusion of granulocyte colony‐stimulating factor,^6^ lack sufficient evidence to support their widespread use. Therefore, the management of refractory thin endometrium that does not respond to standard therapies during ART programs remains a significant challenge.

In 2015, Chang et al.^7^ reported remarkable improvements in reproductive outcomes in patients with a thin endometrium following an infusion of intrauterine platelet‐rich plasma (PRP) for the first time. Activated platelets secrete growth factors such as vascular endothelial growth factor, transforming growth factor, platelet‐derived growth factor, and epidermal growth factor, which stimulate cell proliferation, angiogenesis, and subsequent tissue regeneration. Because autologous PRP is derived from the patients' own fresh whole blood, its administration is associated with no serious side effects, such as rejection reactions. Consequently, PRP is widely used in various therapeutic fields. Intrauterine PRP infusion was initially administered to patients with a thin endometrium,^7^, ^8^, ^9^, ^10^, ^11^, ^12^ followed by those with RIF^13^, ^14^, ^15^, ^16^ or chronic endometritis.^17^ Although the detailed mechanisms underlying the effects of PRP on the endometrium remain unclear, several recent studies and meta‐analyses have demonstrated the beneficial effects of an intrauterine PRP infusion.^18^, ^19^, ^20^, ^21^, ^22^ In this retrospective study, we aimed to identify factors that influence the reproductive outcomes of the intrauterine PRP infusion in frozen and thawed embryo transfer (FET) programs in patients with thin endometrium and/or a history of RIF.

## 2 MATERIALS AND METHODS

### 2.1 Study sample

We enrolled infertile patients with either a thin endometrium and/or RIF who underwent FET followed by the first intrauterine PRP infusion at our clinic between August 2020 and December 2022. Patients with a thin endometrium were defined as those with a persistently thin endometrium (<8 mm) during at least the two previous hormone replacement cycles that resulted in the failure of implantation, despite the transfer of good‐quality blastocysts (Gardner grade 4BB or higher), or led to FET cancellation. Patients with RIF were characterized by a history of implantation failure during at least two consecutive FET cycles involving good‐quality blastocysts (Gardner grade 4BB or higher), but without thinning of the endometrium. All the eligible participants had at least one remaining frozen blastocyst (Gardner grade 4BB or higher), were provided with comprehensive information, regarding the PRP treatment before commencing their FET cycle, and made their own decisions regarding whether to undergo PRP therapy. Written informed consent for blood sampling, intrauterine PRP infusion, and inclusion in the present study was obtained from all the participants.

Before participating in this study, all the participants had undergone hysteroscopic examination to confirm the absence of anatomical abnormalities and underwent blood tests for antiphospholipid antibody syndrome (APS). Moreover, endometrial receptivity analysis (ERA; ©Igenomix, Valencia, Spain) and endometrial microbiome metagenomic analysis (EMMA; ©Igenomix, Valencia, Spain) were performed in all the participants with RIF and in the participants with thin endometrium who requested these analyses. Patients who generated abnormal results in these tests underwent at least one additional FET cycle but did not achieve pregnancy, even after appropriate treatment. These additional treatments included personalized FET with a modified transfer day for the treatment of non‐receptive ERA, symbiotics with/without antibiotics for the modification of non‐*Lactobacillus*‐dominant microbiota, defined as the proportion of *Lactobacillus* in the endometrial microbiome <90%, or anticoagulants (low‐dose aspirin and/or heparin) for participants with positive antiphospholipid antibodies (APA).

The exclusion criteria for PRP treatment included hemoglobin <11 g/dL; platelet count <15 million/mm^3^; blood‐borne diseases such as hepatitis B, hepatitis C, syphilis, and human immunodeficiency virus infection; and active lower genital tract infection. Patients who were undergoing other conventional treatments for thin endometrium were not included in this study, and the use of nonsteroidal anti‐inflammatory drugs was prohibited after the conclusion of menstrual bleeding during the treatment cycle.

### 2.2 Endometrial preparation, PRP infusion, and FET

FET with intrauterine PRP infusion was performed during a hormone replacement cycle. The endometrial preparation involved the use of transdermal estradiol tape (Estrana®, 2.16 mg every 2 days; Hisamitsu Pharmaceutical Co., Inc., Saga, Japan), which was initiated on day 4 of the cycle. Intrauterine PRP infusion was performed twice per cycle, on the 9th to 11th days of the cycle, and 2 days later. Autologous PRP was prepared on the day of PRP infusion. Briefly, 20 mL of venous blood was drawn into two Acti‐PRP tubes (Aeon Biotherapeutics Inc., Taipei, Taiwan) and centrifuged at 2000 × *g* for 6 min. The upper layer containing platelet‐poor plasma was discarded, and the remaining platelet‐rich plasma was mixed with the buffy coat to generate PRP. Approximately 1.0 mL of PRP was aspirated into a 2.5 mL syringe and slowly infused into the uterine cavity using an elastomer catheter for intrauterine insemination (Sankyo Medic Co., Ltd., Shizuoka, Japan). Bed rest was not required after the procedure.

A transvaginal ultrasonographic examination was performed on the days of PRP injection and 2–4 days afterward to assess the endometrium and ovaries. If the endometrium exhibited a trilaminar appearance and a thickness of >8 mm, exogenous progesterone supplementation was initiated *per vaginam* using Luteum® vaginal suppositories (400 mg twice daily; ASKA Pharmaceutical Co., Ltd., Tokyo, Japan). For the participants who had an endometrial thickness of <8 mm, even after ≥10 days following PRP infusion, those with a trilaminar endometrial appearance underwent embryo transfer, irrespective of their endometrial thickness, whereas those with a thin endometrium that appeared abnormal had their FET canceled. A single good‐quality frozen blastocyst was thawed and transferred to the uterine cavity 5–6 days after the initial progesterone administration, as guided by the ERA results. The quality of the transferred embryos was assessed by the Gardner grade of thawed embryos at the time of transfer, and the developmental speed of embryos, which was represented by the number of days from fertilization to freezing when embryos had reached expanded blastocysts.

The serum concentration of β‐hCG were measured 10–12 and 14–16 days after the initial administration of progesterone in our laboratory using the Access 2 immunoassay system (Beckman Coulter, Brea, CA, USA). The serum β‐hCG concentration was considered to be positive if it exceeded 5 mIU/mL on two occasions, ≥10 days following the initial administration of progesterone, and clinical pregnancy was confirmed by identifying a gestational sac on transvaginal ultrasonography. Exogenous supplementation with estradiol and progesterone continued until the eighth week of gestation.

### 2.3 Statistical analysis

All statistical analyses were conducted using the JMP software v.17.1.0 (SAS Institute Inc., Cary, NC, USA). We used Student's *t*‐test and analysis of variance to compare normally distributed datasets, whereas categorical datasets were compared using Pearson's chi‐square test. Single and multivariate logistic regression analyses were performed to identify predictive factors influencing the therapeutic outcomes of PRP‐FET, including β‐hCG positivity, clinical pregnancy, and live birth. The explanatory variables were chosen using the bootstrap forest platform provided by the JMP software. Odds ratios and 95% confidence intervals were calculated, and the Wald test was used to assess the overall relationship. Trend analyses of the relationship between ordinal variables representing the number of previous implantation failures and the therapeutic outcomes were performed using the Cochran–Armitage trend test. All the tests were two‐tailed, and statistical significance was accepted at *p* < 0.05.

## 3 RESULTS

**FIGURE 1:**
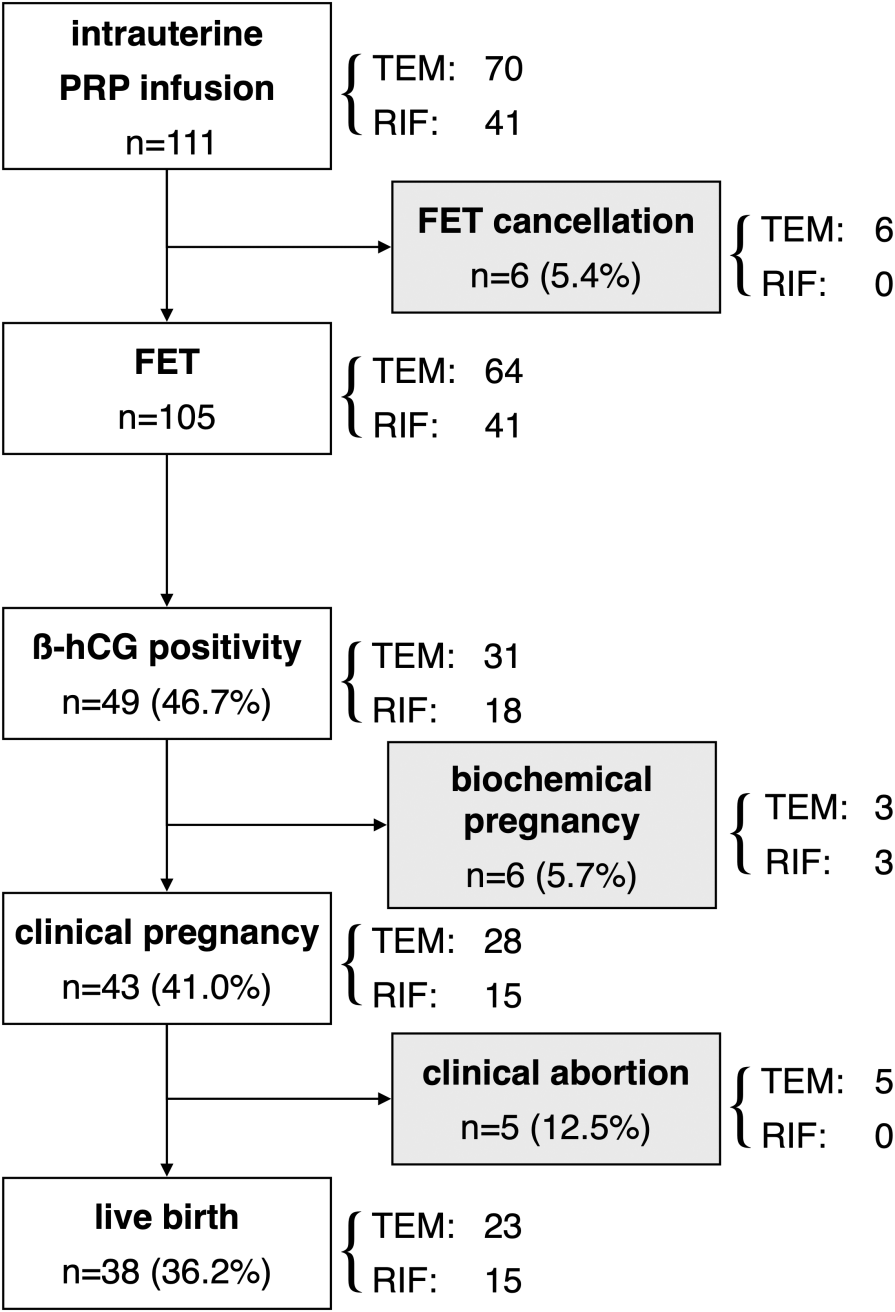
Diagram showing the participation of patients throughout the study. The numbers of participants are shown. Intrauterine PRP infusion was administered to 111 participants. No one met the exclusion criteria. FET cancellation occurred in six patients. Among 105 cycles of FET, biochemical pregnancies occurred in six cycles and clinical pregnancies occurred in 43 cycles, of which five resulted in clinical abortions, and 38 live births were achieved. FET, frozen–thawed embryo transfer; PRP, platelet‐rich plasma; RIF, recurrent implantation failure; TEM, thin endometrium; β‐hCG, beta‐human chorionic gonadotropin.

We recruited 111 eligible patients aged 28–47 years and studied 111 cycles (Figure 1). None of the patients met the exclusion criteria. The participants had previously undergone a total of 406 unsuccessful FET cycles, resulting in an overall implantation failure rate of 91.1% (370/406). The biochemical pregnancy rate was 7.6% (31/406), the clinical pregnancy rate (CPR) was 8.9% (36/406), and there were no live births (LBR, 0%). The indications for PRP infusion were a thin endometrium in 70 participants (the TEM group) and RIF without a thin endometrium in 41 participants (the RIF group). Comparison of these two groups revealed no significant differences in age, body mass index (BMI), the duration of infertility, the cause of infertility, the reproductive outcomes of the previous embryo transfers (ETs), the proportion of abnormal results on ERA/EMMA, or the prevalence of positivity for APA. However, the RIF group had a larger incidence of previous implantation failure. In the TEM group, six FET cycles were canceled, owing to the presence of a thin and abnormal‐appearing endometrium due to abnormal uterine bleeding. Of the 105 FET cycles that proceeded, the β‐hCG positivity rate (hCGR) was 46.7% (49/105), the CPR was 41.0% (43/105), and the LBR was 36.2% (38/105). The CPR and LBR were significantly higher than during previous FET cycles. However, there were no significant differences in the hCGR, CPR, or LBR between the TEM and RIF groups (Table 1). Of the 43 clinical pregnancies achieved, five (11.6%) ended in miscarriage, all of which occurred in the TEM group, with four of these being attributed to chromosomal aneuploidy.

**TABLE 1:** Characteristics of the participants and the results of intrauterine PRP infusion in the TEM and RIF groups.

	Total	(*n* = 111)	TEM	(*n* = 70)	RIF	(*n* = 41)	*p*
	*M* ± SD		*M* ± SE	95% CI	*M* ± SE	95% CI	
Age (year)	38.2 ± 4.4		38.3 ± 0.5	37.3–39.4	37.9 ± 0.7	36.6–39.2	0.6427
BMI (kg/m^2^)	22.3 ± 3.4		22.5 ± 0.4	21.7–23.3	21.9 ± 0.5	20.8–22.9	0.3489
Duration of infertility (year)	3.1 ± 2.0		3.1 ± 0.2	2.7–3.6	3.1 ± 0.3	2.5–3.7	0.8667
No. of previous implantation failure	3.3 ± 2.6		2.8 ± 0.3	2.2–3.4	4.2 ± 0.4	3.5–5.0	0.0047

	*n*	%	*n*	%	*n*	%	
Cause of infertility (excluding thin endometrium)	111		70		41		
Ovulation	9	8.1	6	8.6	3	7.3	0.6992
Tubal	17	15.3	13	18.6	4	9.8	
Male	20	18.0	12	17.1	8	19.5	
Complex	7	6.3	3	4.3	4	9.8	
Unexplained	58	52.3	36	51.4	22	53.7	
Outcomes of previous ET (cycle)	406		216		190		
Biochemical pregnancy (% per ET)	31	7.6	13	6.0	18	9.5	0.4250
Clinical pregnancy (% per ET)	36	8.9	19	8.8	17	8.9	0.9985
Live birth (% per ET)	0	0	0	0	0	0	–
Implantation failure (% per ET)	370	91.1	197	91.2	173	91.1	0.9986
No. of non‐receptive ERA	34/66	51.5	12/24	50.0	22	53.7	0.9602
No. of non‐*Lactobacillus* dominant EMMA	43/66	65.2	17/24	70.8	26	63.4	0.8302
No. of positive for antiphospholipid antibodies	17	15.3	7/69	10.1	10	24.4	0.1269
FET cancellation (% per PRP)	6	5.4	6	8.6	0	0	0.1561
Outcomes of PRP‐FET (cycle)	105		64		41		
β‐hCG positivity (% per ET)	49	46.7	31	48.4	18	43.9	0.6495
Biochemical pregnancy (% per ET)	6	5.7	3	4.7	3	7.3	0.8518
Clinical pregnancy (% per ET)	43	41.0	28	43.8	15	36.6	0.4664
Spontaneous abortion (% per clinical pregnancy)	5	11.6	5	17.9	0	0	0.1860
Live birth (% per ET)	38	36.2	23	35.9	15	36.6	0.9463

*Note*: Continuous datasets were compared between the TE and RIF groups using Student's *t*‐test, and categorical datasets were compared using Pearson's chi‐square test. Non‐*Lactobacillus* dominance was defined as the proportion of *Lactobacillus* in the endometrial microbiome of <90%. Six FET cancellations were due to abnormal uterine bleeding that caused a thin and abnormal endometrium.

Abbreviations: BMI, body mass index; CI, confidence interval; EMMA, endometrial microbiome metagenomic analysis; ERA, endometrial receptivity analysis; ET, embryo transfer; FET, frozen–thawed embryo transfer; *M*, mean; PRP, platelet‐rich plasma; RIF, recurrent implantation failure; SD, standard deviation; SE, standard error of the mean; TEM, thin endometrium.

Although the mean endometrial thickness throughout the PRP‐FET cycles was lower in the TEM group than in the RIF group, the TEM group achieved a greater thickness during the PRP‐FET cycle than during the previous hormone replacement cycle (Table 2). The mean increased ratio of final endometrial thickness on the day of initial progesterone administration compared to the previous failed cycle or before the first PRP infusion was significantly higher in the TEM group than in the RIF group. However, there was no difference in these ratios between participants who achieved clinical pregnancy and those whose pregnancies failed in either the TEM or RIF groups during the treatment cycle. Moreover, the Gardner grade of transferred embryos and the number of days after fertilization when the transferred embryo was frozen did not differ between participants who achieved clinical pregnancy and those whose pregnancies failed in either the TEM or RIF group during the treatment cycle (Table 3).

**TABLE 2:** Changes in endometrial thickness before and after an infusion of PRP in the TEM and RIF groups, according to clinical pregnancy status.

Endometrial thickness (mm)	TEM				RIF			
	Total (*n* = 64)	Success (*n* = 28)	Failure (*n* = 36)	*p*	Total (*n* = 41)	Success (*n* = 15)	Failure (*n* = 26)	*p*
The previous unsuccessful cycle (a)	6.8 ± 0.1*†	7.0 ± 0.1	6.9 ± 0.1	0.4912	9.8 ± 0.1†	9.7 ± 0.3	9.8 ± 0.2	0.7636
The day of first PRP infusion (b)	6.1 ± 0.1‡	6.3 ± 0.2	6.1 ± 0.2	0.2641	8.4 ± 0.2‡	8.3 ± 0.3	8.5 ± 0.2	0.6888
The day of second PRP infusion	7.4 ± 0.2§	7.8 ± 0.2	7.4 ± 0.2	0.1321	9.4 ± 0.2§	9.7 ± 0.4	9.3 ± 0.2	0.3749
The day of initial P administration (c)	8.3 ± 0.2*||	8.4 ± 0.2	8.3 ± 0.2	0.7826	10.1 ± 0.2||	10.2 ± 0.3	10.0 ± 0.2	0.7552
Increased ratio compared to the previous cycle (c/a)	1.10 ± 0.02¶	1.13 ± 0.03	1.08 ± 0.03	0.2749	0.98 ± 0.02¶	1.00 ± 0.03	0.96 ± 0.03	0.2707
Increased ratio compared to before PRP infusion (c/b)	1.38 ± 0.03¶	1.34 ± 0.05	1.40 ± 0.04	0.3596	1.22 ± 0.03¶	1.23 ± 0.04	1.21 ± 0.03	0.6479

*Note*: The mean endometrial thickness of each group was compared using the paired‐samples *t*‐test between the previous unsuccessful cycle and the day of the initial progesterone administration for FET (*p < 0.0001). The mean endometrial thickness between the TEM and RIF groups was compared using Student's *t*‐test († ‡ §||*p* < 0.0001; ¶ *p* < 0.001). The mean endometrial thickness of the successful and failed cycles was compared using Student's *t*‐test. Values are presented as mean ± standard error of the mean.

Abbreviations: P, progesterone; PRP, platelet‐rich plasma; RIF, recurrent implantation failure; TEM, thin endometrium.

**TABLE 3:** Morphology and developmental speed of transferred embryos in the TEM and RIF groups, according to clinical pregnancy status.

	TEM			RIF		
	Success (*n* = 28)	Failure (*n* = 36)	*p*	Success (*n* = 15)	Failure (*n* = 26)	*p*
Gardner grade						
4AA	10 (35.7)	21 (58.3)	0.0523	6 (40.0)	10 (38.5)	0.7014
4AB	8 (28.6)	2 (5.6)		3 (20.0)	3 (11.5)	
4BA	3 (10.7)	2 (5.6)		2 (13.3)	2 (7.7)	
4BB	7 (25.0)	11 (30.6)		4 (26.7)	11 (42.3)	
Days after fertilization when the transferred embryo was frozen						
D + 5	20 (71.4)	22 (61.1)	0.4912	9 (60.0)	11 (42.3)	0.2866
D + 6	8 (28.6)	14 (38.9)		6 (40.0)	15 (57.7)	

*Note*: Categorical datasets were compared using Pearson's chi‐square test.

Abbreviations: RIF, recurrent implantation failure; TEM, thin endometrium.

**FIGURE 2:**
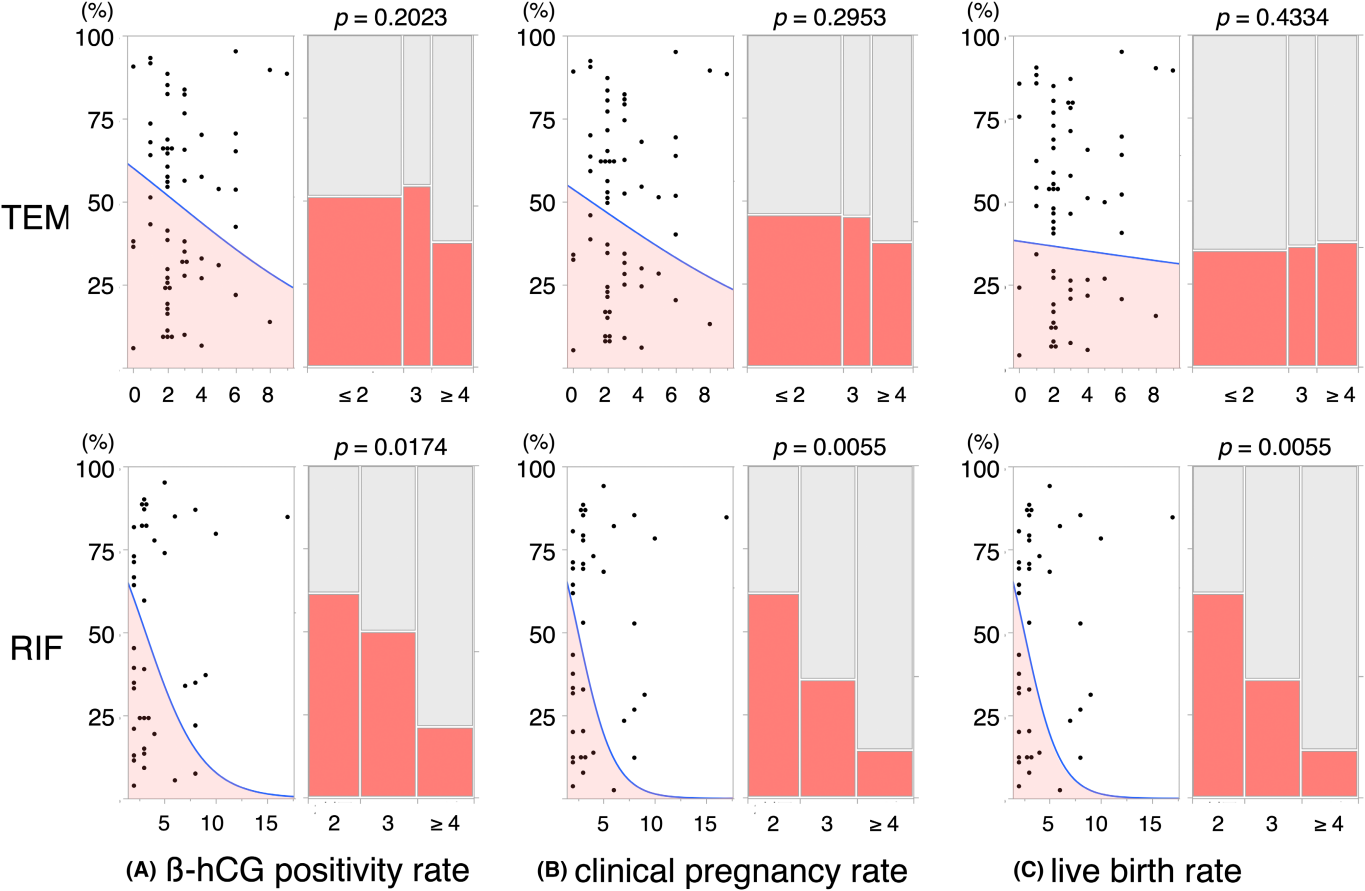
Decreases in the prevalence of successful PRP‐FET alongside increases in the number of previous implantation failures. The graphs depict the relationships of the number of previous implantation failures with the β‐hCG positivity rate (A), clinical pregnancy rate (B), and live birth rate (C) of the TEM (upper) and RIF (lower) groups. Each dot‐plotted graph on the left of the panels represents the outcome of a single logistic regression analysis of the relationship of the number of previous implantation failures with therapeutic outcomes. The colored areas represent the calculated prevalence of positive outcomes. Each bar graph on the right side represents the results of a Cochran–Armitage trend test for the relationship between the number of previous implantation failures and the therapeutic outcome, and the width of each bar indicates the number of participants. The *p*‐values represent the results of Wald tests.

Multivariate logistic regression analyses were performed to identify factors contributing to β‐hCG positivity, clinical pregnancy, and live births using the following explanatory variables: age, BMI, number of previous implantation failures, final endometrial thickness, and Gardner grade for blastocysts. In the TEM group, no factors were associated with β‐hCG positivity or clinical pregnancy, although BMI was weakly associated with live births. However, in the RIF group, the number of previous implantation failures was significantly associated with β‐hCG positivity, clinical pregnancy, and live birth (Table 4). In addition to single logistic regression analyses, Cochran–Armitage trend tests revealed that the number of previous implantation failures was a significant parameter associated with hCGR, CPR, and LBR in the RIF group, but not in the TEM group (Figure 2).

**TABLE 4:** Factors associated with the success of PRP‐FET in the TEM and RIF groups.

	TEM					RIF				
	Crude		Adjusted			Crude		Adjusted		
	OR	*p*	OR	95% CI	*p*	OR	*p*	OR	95% CI	*p*
β‐hCG positivity										
Age (year)	0.94	0.2905	0.97	0.86–1.09	0.5733	0.97	0.6581	0.96	0.91–1.45	0.6565
BMI (kg/m^2^)	0.85	0.0410	0.84	0.71–0.99	0.0462	1.18	0.1689	1.15	0.91–1.54	0.2950
No. of previous implantation failure	0.85	0.2225	0.85	0.62–1.12	0.2577	0.70	0.0460	0.67	0.42–0.94	0.0448
Final endometrial thickness (mm)	0.94	0.7489	0.97	0.62–1.51	0.8833	1.18	0.5447	1.39	0.70–2.93	0.3529
Gardner grade of transferred embryo	0.92	0.6760	0.87	0.57–1.32	0.5206	1.32	0.2482	1.15	0.66–1.15	0.6131
Clinical pregnancy										
Age (year)	0.95	0.3754	0.97	0.86–1.08	0.5651	1.02	0.8035	1.07	0.88–1.34	0.4927
BMI (kg/m^2^)	0.89	0.1392	0.89	0.75–1.04	0.1526	1.08	0.4386	1.02	0.81–1.31	0.8818
No. of previous implantation failure	0.87	0.3018	0.86	0.63–1.13	0.3053	0.56	0.0366	0.50	0.23–0.81	0.0242
Final endometrial thickness (mm)	1.06	0.7786	1.11	0.71–1.08	0.6459	1.11	0.7175	1.66	0.79–4.02	0.2005
Gardner grade of transferred embryo	0.90	0.6084	0.87	0.58–1.31	0.5167	1.69	0.5283	0.93	0.52–1.64	0.8062
Live birth										
Age (year)	0.88	0.0365	0.89	0.66–1.00	0.0588	1.02	0.8035	1.07	0.88–1.34	0.4927
BMI (kg/m^2^)	0.83	0.0339	0.82	0.66–0.98	0.0424	1.08	0.4386	1.02	0.81–1.31	0.8818
No. of previous implantation failure	0.97	0.8130	0.98	0.72–1.31	0.9125	0.56	0.0366	0.50	0.23–0.81	0.0242
Final endometrial thickness (mm)	1.04	0.8705	1.09	0.67–1.76	0.7242	1.11	0.7175	1.66	0.79–4.02	0.2005
Gardner grade of transferred embryo	0.80	0.2773	0.75	0.48–1.17	0.2068	1.69	0.5283	0.93	0.52–1.64	0.8062

*Note*: The crude data were generated using single logistic regression analyses and the adjusted data were generated using multivariate logistic regression analysis. The *p*‐values represent the results of Wald tests.

Abbreviations: BMI, body mass index; CI, confidence interval; FET, frozen–thawed embryo transfer; OR, odds ratio; PRP, platelet‐rich plasma; RIF, recurrent implantation failure; TEM, thin endometrium.

## 4 DISCUSSION

In this retrospective study of patients with a thin endometrium or RIF who underwent hysteroscopy, ERA, EMMA, and blood testing for APS, the reproductive outcomes of FET following intrauterine PRP infusion were significantly better than the previous ART outcomes achieved without PRP infusion. These superior outcomes were achieved consistently in patients with either a thin endometrium or RIF. Although PRP infusion increased endometrial thickness, the final thickness as well as the increased ratio compared with the thickness during previous unsuccessful cycles or before PRP infusion were not associated with pregnancy. In addition, we could not confirm the effect of the quality of transferred embryos on the success or failure of PRP‐FET, under the condition of using good‐quality embryos of 4BB or higher that had reached expanded blastocysts on the fifth to sixth day after fertilization. However, notably, the number of previous implantation failures was closely associated with unsuccessful PRP treatment in patients with RIF but without a thin endometrium. The odds ratio for successful PRP‐FET in patients with RIF decreased by 0.56‐fold with each additional prior implantation failure.

The endometrial thicknesses of the participants following PRP infusion was significantly greater than those during their previous hormone replacement cycles. However, the likelihood of successful embryo implantation was not associated with endometrial thickness, even in the thin endometrium group. There have been suggestions that the crucial effect of PRP on the endometrium may be predominantly related to its functional, rather than its structural, characteristics. For instance, Kuroda et al. demonstrated that PRP promoted the expression of genes associated with cell growth, tissue regeneration, and proinflammatory responses in undifferentiated endometrial stromal cells, while modulating the expression of genes involved in cell proliferation and inflammation in decidualized cells by inhibiting phosphoinositide 3‐kinase signaling.^23^ These findings suggest that the effectiveness of PRP may be consistent among protocols involving differing timings of infusion. A recent meta‐analysis of both randomized controlled trials (RCTs) and non‐RCTs conducted by Maged et al.^22^ showed a beneficial effect of PRP administration on the implantation rate, clinical pregnancy rate, ongoing live birth rate, and endometrial thickness, despite variations in the therapeutic protocols used. A recent open‐label RCT also demonstrated that PRP significantly improves the clinical pregnancy rate, as well as increasing endometrial thickness and vascularity.^24^ However, the ESHRE Working Group on RIF stated that the current evidence is insufficient to fully justify the use of PRP infusion, although its efficacy regarding the prevention of implantation failure warrants further evaluation.^25^ There are also negative studies on the effectiveness of PRP.^26^, ^27^ In those negative studies, citric acid or acid‐citrate‐dextrose was used as an anticoagulant to prepare PRP. It has been suggested that PRP quality, such as cell counts or concentrations of growth factors, might be influenced by the type of anticoagulant used.^28^, ^29^ The Acti‐PRP tubes that we used does not require anticoagulants or additives during PRP preparation. It is necessary to evaluate the effect of these anticoagulants on the action of growth factors and the implantation milieu.

The two primary indications for PRP infusion, a thin endometrium and RIF, are the subject of ongoing debate and disagreement regarding their precise definitions. With respect to a thin endometrium, most studies have used a cutoff of <7 mm or <8 mm. However, there have only been a few studies of endometrial thickness during the FET cycle in the context of hormone replacement therapy. One study showed that patients with an endometrial thickness of <8 mm during their first FET cycle had significantly lower clinical pregnancy and live birth rates.^30^ Another retrospective study of the Canadian IVF database demonstrated that the live birth rates decreases with each millimeter decline in endometrial thickness below 7 mm.^6^ However, two studies of recipients of oocyte donation accompanied by hormone replacement cycles showed that endometrial thickness does not affect pregnancy rate.^31^, ^32^

The debate regarding the definition of RIF has primarily been centered on the number of failed treatment cycles and the number of embryos transferred. Although it is common to define RIF as three or more consecutive failed cycles of embryo transfer using good‐quality embryos, some experts prefer a higher threshold, to avoid false‐positive diagnoses and overtreatment.^33^, ^34^ However, others argue that defining RIF as two consecutive failures may allow for the earlier identification of potential implantation issues and more timely intervention.^35^ A recent comprehensive survey of the definitions in use suggested that a consensus is emerging, in which RIF is defined as the failure to achieve clinical pregnancy after two‐to‐three transfers of good‐quality embryos, while also considering the patient's age.^36^

In this study, we found that the effectiveness of PRP was significantly lower when there had been three or more previous implantation failures. In other words, if treatment is delayed until three implantation failures occur, there may be a risk that a woman misses the opportunity to become pregnant. However, it remains challenging to explain why a larger number of previous implantation failures is associated with a lower likelihood of successful PRP treatment. Pregnancy requires overcoming numerous obstacles, including variations in endometrial receptivity. Most defects in endometrial receptivity typically reduce the chance of successful implantation and potentially prolong the time for women to achieve pregnancy. However, patients with a history of multiple implantation failures may have more significant or severe underlying issues that make the successful achievement of pregnancy following PRP infusion less likely. Lin et al. reported that the number of previous uterine surgeries affected the efficacy of PRP in terms of the successful pregnancy rate.^37^ In this study, two patients with a thin endometrium group had extensive and severe damage to the basal layer of the endometrium, which was likely the result of previous surgical procedures, such as hysteroscopic surgery using electrical devices or repeated curettages. These injuries proved to be resistant to PRP infusion and ultimately led to cancellation of the FET. Such irreversible defects in endometrial stem cells, which are responsible for regeneration, may necessitate the use of alternative treatments to PRP.

Because the cost of PRP therapy is not subsidized in Japan, it is associated with a significant economic burden for patients. Despite the growing recognition that PRP may represent a novel treatment option for intractable cases, it is often assigned a lower priority than other adjuvants. Indeed, it is inevitable that interventions that are eligible for insurance coverage or subsidization will be used in preference. A recent review regarding RIF recommended the assessment of APS in women with RIF,^25^ owing to their higher risk of having any type of APA.^38^ Another emerging issue regarding the use of adjuvants and endometrial receptivity is the role of the microbiome, which may also influence the risk of implantation failure.^39^ Previously, we reported that the absence of *Lactobacillus* from the endometrial microbiota might delay the development of endometrial receptivity.^40^ In this study, the prevalences of non‐receptivity in ERA, non‐*Lactobacillus*‐dominant microbiota in EMMA, and APA positivity were similar to those of infertile patients attending our clinic. Although it seems unlikely that women with a thin endometrium or RIF are more likely to have abnormal results of these tests, the management of abnormal test results may increase the success of intrauterine PRP infusion. Preimplantation genetic testing (PGT) is a highly reliable method of detecting and ruling out embryonic defects, although the potential for embryo biopsies to cause significant damage cannot be ignored. In this study, when PGT was not performed, four of the five miscarriages that occurred in participants with a thin endometrium and who were aged 38–47 years were associated with chromosomal aneuploidy of the embryo. Therefore, clinicians should consider performing PGT before administering a PRP infusion in patients who have already planned to undergo FET.

Although the choice of adjuvant depends on patient preference and regional conditions, further investigation is necessary to provide more well‐founded clinical management strategies. A proposal has been made to initiate further investigations to identify RIF if the predicted values of cumulative implantation reached 60%.^25^ It has also been suggested that RIF should not be diagnosed until at least three implantation failures following euploid embryo transfer, and that more failures should be accepted if unscreened embryo transfers are used, with adjustment for the age of patients.^41^ In the clinical context, the criteria for investigating implantation failure should not be overly stringent, because this could significantly affect the prognosis of patients with RIF. For women with poor ovarian reserves and where there is a low chance of obtaining good‐quality embryos, it may be unacceptable to continue the same standard treatment for up to three cycles. Thus, it is imperative to discuss whether two consecutive ET implantation failures warrant further intervention, including intrauterine PRP infusion.

In this study, we have found that the intrauterine infusion of PRP increases endometrial thickness and improves pregnancy outcomes in patients with RIF or a thin endometrium. In addition, we identified a higher successful implantation rate following PRP infusion in patients with RIF who had experienced fewer than three previous implantation failures. The limitations of this study include its small size, retrospective design, single‐center nature, and selection bias due to recruitment based on the willingness of patients to participate without considering patient age. Moreover, we defined thin endometrium as less than 8 mm, although some studies have reported that a definition of less than 7 mm is better for predicting reproductive success. It should be noted that conclusions may differ depending on the definition. However, we believe that the findings of this study are reliable because the study was conducted using a consistent approach in a standardized environment, thereby controlling for the presence of other factors that could have contributed to implantation failure, with the exception of embryo aneuploidy. Based on these findings, intrauterine PRP infusion should be considered beneficial for patients who have experienced failure of two FETs using good‐quality blastocysts.

In summary, intrauterine PRP infusion is a safe and promising treatment option that is easily accessible and relatively noninvasive for patients undergoing ART. The present findings may assist health care professionals in deciding whether PRP infusion should be used as a treatment in refractory cases. However, further research is necessary to enhance our understanding of the therapeutic benefits of intrauterine PRP infusion in patients who experience implantation failure.

## CONFLICT OF INTEREST STATEMENT

The authors declare no conflict of interest.

## ETHICS STATEMENT

This study was approved by the Certified Committee for Regenerative Medicine (accreditation No. NA8160008) and submitted to the Ministry of Health, Labour, and Welfare in Japan (Clinical Study No. PB2200002). The study protocol was approved by the Ethics Committee of the clinic.

## HUMAN RIGHTS STATEMENTS AND INFORMED CONSENT

All procedures followed were in accordance with the ethical standards of the responsible committee on human experimentation (*institutional and national*) and the Helsinki Declaration of 1964 and its later amendments. Informed consent was obtained from all patients for being included in this study.

## Data Availability

The participants in this study did not provide written consent for their data to be publicly shared. Owing to the sensitive nature of this research, supporting data are not available.
